# Rheumatic mitral stenosis in Children: more accelerated course in sub-Saharan Patients

**DOI:** 10.1186/1471-2261-13-95

**Published:** 2013-11-01

**Authors:** Henok Tadele, Wubegzier Mekonnen, Endale Tefera

**Affiliations:** 1 Department of Pediatrics & Child Health, School of Medicine, Hawassa University, Hawassa, Ethiopia; 2 Department of Reproductive Health and Health Service Management, School of Public Health, Addis Ababa University, Addis Ababa, Ethiopia; 3 Department of Pediatrics & Child Health, Addis Ababa University and Cardiac Center Ethiopia, Addis Ababa, Ethiopia

**Keywords:** Mitral stenosis, Valve area, Rheumatic heart disease, Sub-Saharan Africa

## Abstract

**Background:**

Mitral stenosis, one of the grave consequences of rheumatic heart disease, was generally considered to take decades to evolve. However, several studies from the developing countries have shown that mitral stenosis follows a different course from that seen in the developed countries. This study reports the prevalence, severity and common complications of mitral stenosis in the first and early second decades of life among children referred to a tertiary center for intervention.

**Methods:**

Medical records of 365 patients aged less than 16 and diagnosed with rheumatic heart disease were reviewed. Mitral stenosis was graded as severe (mitral valve area < 1.0 cm^2^), moderate (mitral valve area 1.0-1.5 cm^2^) and mild (mitral valve area > 1.5 cm^2^).

**Results:**

Mean age at diagnosis was 10.1 ± 2.5 (range 3–15) years. Of the 365 patients, 126 (34.5%) were found to have mitral stenosis by echocardiographic criteria. Among children between 6–10 years, the prevalence of mitral stenosis was 26.5%. Mean mitral valve area (n = 126) was 1.1 ± 0.5 cm^2^ (range 0.4-2.0 cm^2^). Pure mitral stenosis was present in 35 children. Overall, multi-valvular involvement was present in 330 (90.4%). NYHA functional class was II in 76% and class III or IV in 22%. Only 25% of patients remember having symptoms of acute rheumatic fever. Complications at the time of referral include 16 cases of atrial fibrillation, 8 cases of spontaneous echo contrast in the left atrium, 2 cases of left atrial thrombus, 4 cases of thrombo-embolic events, 2 cases of septic emboli and 3 cases of airway compression by a giant left atrium.

**Conclusion:**

Rheumatic mitral stenosis is common in the first and early second decades of life in Ethiopia. The course appeared to be accelerated resulting in complications and disability early in life. Echocardiography-based screening programs are needed to estimate the prevalence and to provide support for strengthening primary and secondary prevention programs.

## Background

Though rare in developed countries [1-3], Rheumatic Heart Disease (RHD) continues to be a serious health problem in the developing countries [4,5]. RHD in these much of the world has not declined [6]. Recent echocardiography-based surveys in some developing countries have estimated the prevalence of RHD to be 3–10 times compared to previous estimates based on clinical examination alone [7-10].

Unlike other valvular lesions, which might be attributed to multiple etiologies, mitral stenosis alone or in combination with other valvular lesions is almost exclusively attributed to RHD [11,12]. Congenital mitral stenosis is an exceedingly rare form of mitral stenosis that is associated with serious circulatory disturbance and high mortality within the first few years of life [13-15].

The severity of rheumatic mitral valve disease in the developing countries differs in many ways from that in the industrialized countries [16]. In studies from developed countries mitral stenosis was considered a delayed manifestation [17] and less common especially in the first decade of life suggesting that it takes several decades to evolve [11,18,19]. This inference was supported by echocardiography-based longitudinal studies that have estimated the average decline in valve area to be as low as 0.09 cm^2^/year [20,21].

In contrast, studies from developing countries document rapid progression of mitral stenosis leading to serious disability early in life that requires treatment [22-27]. In developing regions, predisposing factors to recurrent rheumatic fever persist, prophylactic penicillin is often not available and disease progression is not detected [24,28]. Patients in most areas of the developing world, including those who know their diagnosis, do not receive the secondary prophylaxis [6,29].

Sub-Saharan Africa (where illiteracy is rampant, access to medical care is scarce and echocardiography based screening practices are less feasible) would be expected to have a high prevalence of RHD. Ethiopia has one of the highest estimated prevalence of RHD in the world [30], but echocardiography-based studies in children are not available. This study reports the prevalence and severity of mitral stenosis in children aged <16 years who were referred to a tertiary academic center for treatment.

## Methods

Medical records of all patients with rheumatic heart disease who were referred to the cardiac center in Addis Ababa, between its opening in January 2009 to December 2012 were reviewed. Patients were included in the study if their age was less than16 years and they had echocardiographic diagnosis of chronic rheumatic valvular heart disease. Patients were excluded if valve area measurement was performed using other methods than area tracing technique (like mean transmitral diastolic pressure gradient technique or pressure-half-time technique) in a setting of associated mitral regurgitation.

Demographic, clinical, electrocardiogram, roentgenogram and echocardiographic data were collected from the patient’s records. Severity of mitral stenosis was graded as mild (valve area > 1.5 cm^2^), moderate (valve area 1.0-1.5 cm^2^) and severe (valve area < 1.0 cm^2^). Functional status of the patients was graded according to the New York Heart Association (NYHA) based on the clinical symptoms and signs documented at the time of referral. The ethics committee of the department of pediatrics and child health of the School of Medicine approved the study.

### Statistical methods

Data were first entered into Excel spread sheet. SPSS software version 20 for Windows was used for data analysis. Descriptive statistics were analyzed for baseline variables. Continuous variables were calculated as mean ± SD (range).

## Results

**Figure 1:**
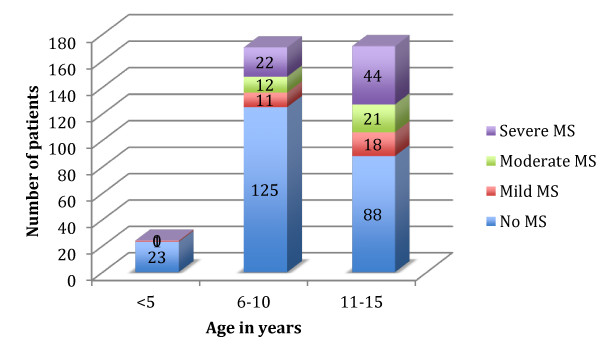
Distribution of mitral stenosis at different ages in children with chronic rheumatic heart disease (n = 365).

During the period of January 2009 to December 2012, a total of 365 children with echocardiographic diagnosis of chronic rheumatic heart disease, and aged <16 years were referred to the cardiac center for possible surgical or percutaneous intervention. Mean age at diagnosis for all patents was 10.1 ± 2.5 (range 3–15) years. Mean body weight for all patients was 26.2 ± 8.2 kg (range 12-48 kg). Of the 365 patients with chronic rheumatic heart disease, 126 (34.5%) had mitral stenosis (Figure 1). Mean mitral valve area for patients with mitral stenosis (n = 126) was 1.1 ± 0.5 cm^2^ (range 0.4-2.0 cm^2^). Their demographic, clinical and echocardiographic characteristics are shown in Table 1.

**Table 1:** Demographic, clinical and echocardiographic characteristics for patients with chronic rheumatic valvular heart disease (n = 365)

**Variables**		**Frequency (percentage)**
Gender (females)		212 (58.1)
Age at diagnosis (years):		
	≤ 5	24 (6.6)
	6–10	170 (46.6)
	11-15	171 (46.8)
Residence (urban or semi-urban):		178 (48.8)
Mitral valve disease		359 (98.4)
Pure mitral stenosis (all grades)		35 (10.0)
Mitral stenosis with regurgitation (all grades)		91 (24.9)
Mitral regurgitation, no stenosis (all grades)		233 (63.8)
Multi-valvular involvement (mitral valve disease included):		
Aortic valve disease (regurgitation and/or stenosis)		257 (70.4)
Tricuspid valve disease (regurgitation and/or stenosis)		299 (81.9)
Mean transmitral diastolic pressure gradient (n = 108):		
	<10 mmHg	4 (3.5)
	10 – 20 mmHg	40 (35.7)
	21 – 30 mmHg	61 (54.5)
	>30 mmHg	3 (2.7)
Mitral valve score for severe mitral stenosis (n = 19)		
	≤8	2 (10.5)
	>8	17 (89.5)
Functional class (New York Heart Association):		
	I	10 (2.7)
	II	276 (75.6)
	III	27 (7.4)
	IV	52 (14.2)
On secondary prophylaxis against recurrence of rheumatic fever (at the time of referral)		272 (74.5)

**Figure 2:**
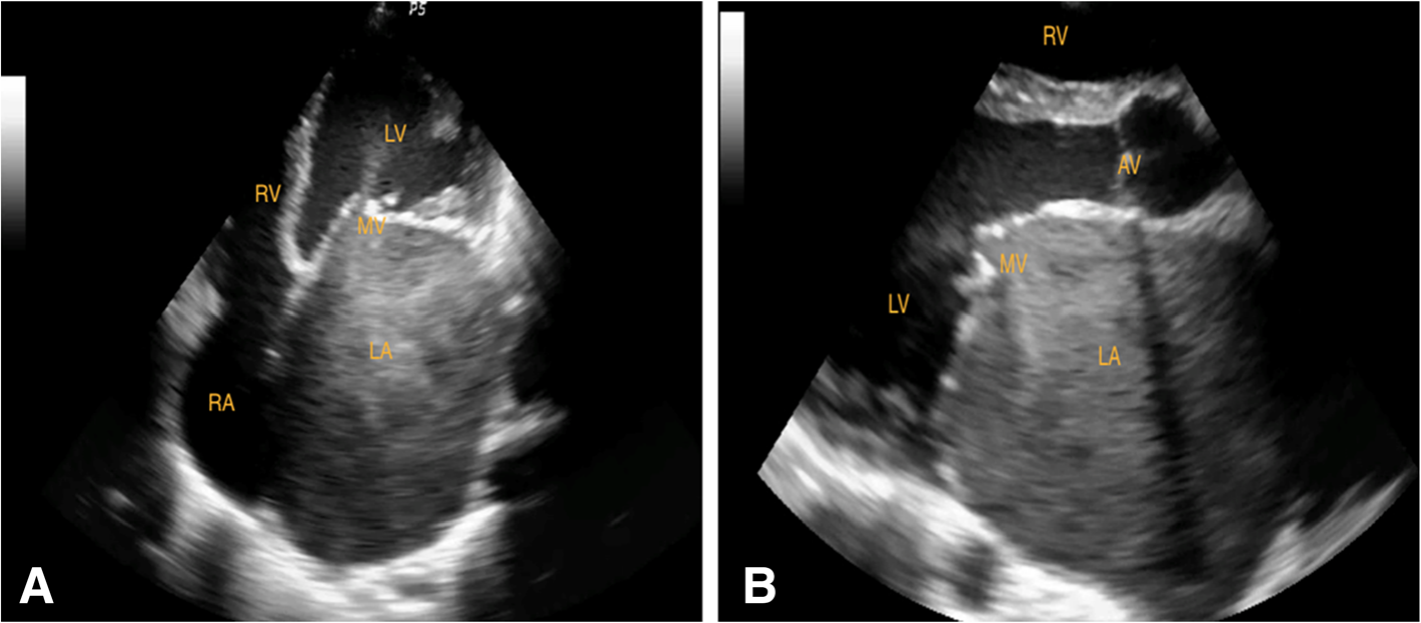
**Echocardiographic frames from a 10-year-old-girl with rheumatic heart disease and severe mitral stenosis (MVA 0.51 cm**^**2**^**), showing enlarged left atrium and spontaneous echo contrast in the left atrium, A. Apical four chamber view (diastole), B. Parasternal long axis view (diastole); LA, left atrium; LV, left ventricle; RA, right atrium; RV, right ventricle; MV, mitral valve; AV, aortic valve.**

**Figure 3:**
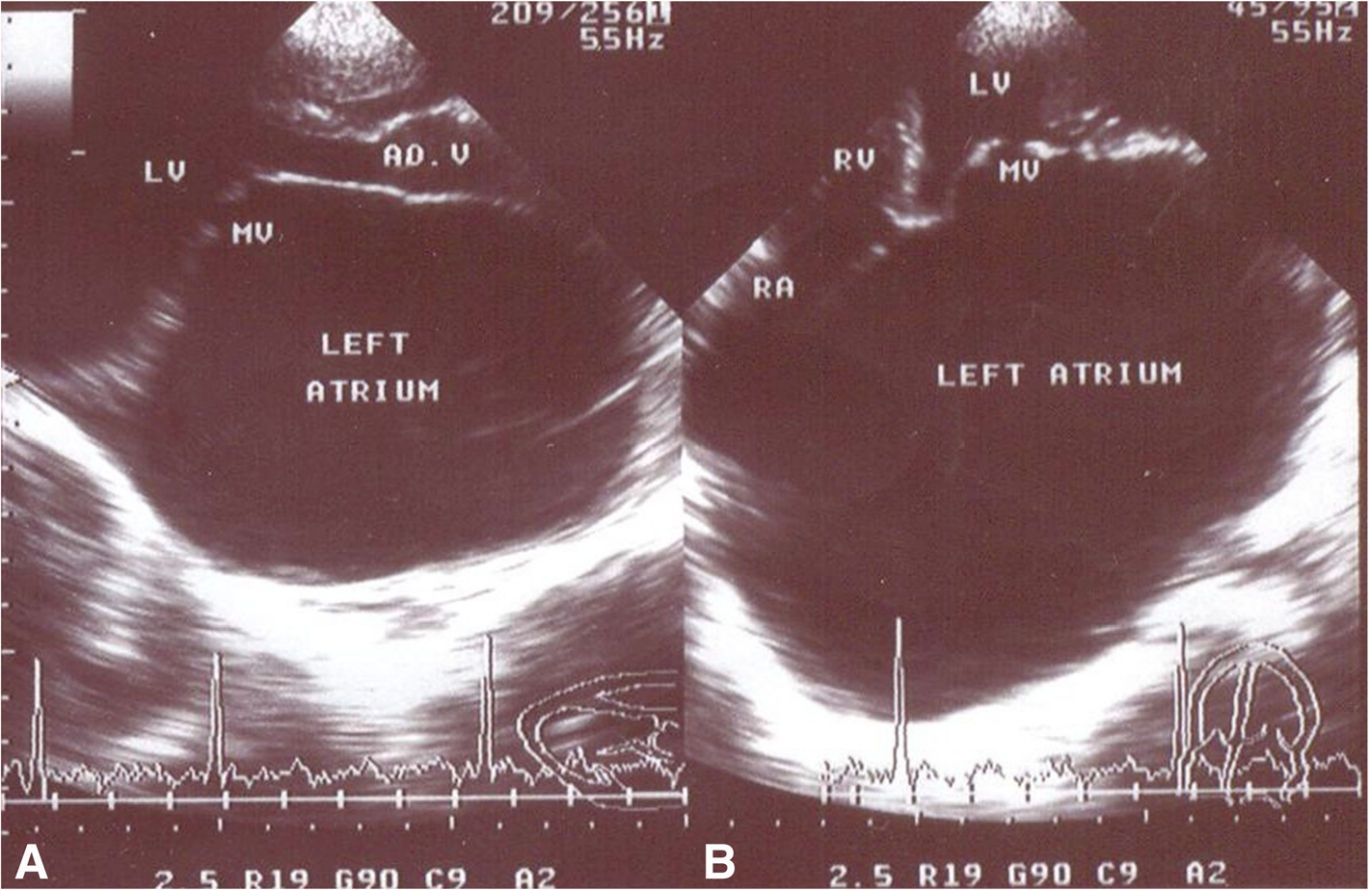
**Echocardiographic frames in the parasternal long axis (A) and apical four chamber (B) views from a 13 year-old-girl with severe mitral stenosis (MVA = 0.84 cm**^**2**^**) and mild regurgitation, showing Giant Left Atrium (GLA).** MV, mitral valve; LV, left ventricle; RV, right ventricle; RA, right atrium.

Two patients with severe mitral stenosis had thrombus in the left atrium. Eight patients with severe mitral stenosis had spontaneous echo contrast in the left atrium (Figure 2). One patient with combined mitral stenosis and regurgitation and two patients with severe mitral regurgitation had evidence of bronchial compression from giant left atrium (GLA) (Figure 3). Pulmonary complications included complete atelectasis of the left lung in one patient with mitral stenosis and a second with severe mitral regurgitation. A third patient had right middle lobe collapse.

Structural tricuspid valve stenosis was present in 19 (5.2%) patients. All patients with tricuspid valve stenosis had severe mitral stenosis. Electrocardiographic evidence of atrial fibrillation was present in 16 (4.4%) of patients. Thrombo-embolic events occurred in 4 patients with severe mitral stenosis, leading to presumed hemiparetic embolic strokes in two patients and gangrene and subsequent amputation of one leg in two patients. Two other patients without mitral stenosis had loss of pulses resulting from presumed septic emboli. Only 91 (24.9%) of the patients remembered having symptoms suggestive of acute rheumatic fever.

## Discussion

In this case series of Ethiopian pediatric patients with RHD, the overall prevalence of mitral stenosis in our patients was 34.5% and the prevalence of severe mitral stenosis, defined as a valve area of less than 1.0 cm^2^, was 18.1%. This figure appears to be high but was difficult to make comparison with other studies, as the study setting and methodology were importantly different from our study. The prevalence of pure mitral stenosis in our series was 9.5%, a rate almost four-fold greater than a similar study reported by Yuko-Jowi et al. from Kenya [31]. The mean trans-mitral diastolic pressure gradient (n = 108) was ≥10 mmHg in 104 (96.5%) of patients and it was greater than 20 mmHg in 64 (57.2%) of the 108 patients. However, this parameter was not used for grading severity of mitral stenosis as most patients had significant associated mitral valve regurgitation that may exaggerate the trans-mitral diastolic gradient.

A striking observation was the high prevalence of mitral stenosis in patients between ages 6–10 years (26.5%). In studies from developed countries mitral stenosis in this age group is rare [11]. Our study confirms those from other developing countries [16,22,23,31,32] suggesting that mitral stenosis can progress rapidly and may lead to severe disability at an early age.

Several reasons could account for the differing course of RHD in underdeveloped countries compared to developed countries. First, predisposing factors to acute rheumatic fever persist and prophylactic penicillin therapy is often inadequate [24]. Second, while secondary prophylaxis can prevent or significantly reduce the development of mitral or aortic valve stenosis [33], many RHD patients lack access or fail to adhere to secondary prophylaxis [6,29]. Patients in developing countries, who adhere to secondary prophylaxis [33,34] have a course similar to those in the developed world. Furthermore, decline in prevalence of rheumatic heart disease itself and even severity of mitral stenosis has paralleled changes in socio-economic factors in some of fastest developing nations in South East Asia [35-37]. The fact most of the patients didn’t remember an attack of rheumatic fever in the past may also contribute to ongoing carditis. Though the role of anti-inflammatory treatment in an acute rheumatic carditis is not well-substantiated, lack of penicillin treatment or bed rest significantly contribute to ongoing carditis [38]. These observations suggest that raising public awareness and improving adherence to primary and secondary prophylaxis could reduce the rates of rheumatic heart disease and its complications.

The low rate of recall of symptomatic episodes consistent with acute RF (24.9%) is consistent with other studies [11,18,39]. The most likely explanation is that acute rheumatic fever escapes attention if it is not associated with migratory polyarthritis or Sydenham’s chorea, especially in medically unsophisticated regions.

Our finding that all patients with tricuspid stenosis have severe mitral stenosis has also been found by another study in the united states [40]. Atrial fibrillation was present in 4.4% of our patients. Other studies have found higher rates ranging between 5.9% and 40% [11,41]. However, considering the younger age of our patients, this percentage is alarming. Rare complications such as airway compression by a giant left atrium (GLA) occurred in one patient with combined mitral stenosis and regurgitation and two other patients with severe mitral regurgitation. The compression has led to complete atelectasis of the left lung in two patients and right middle lobe collapse in the third. This complication has been reported in many case reports, generally, in adults [42-44].

Our patients appeared to have a high rate of secondary prophylaxis against recurrence of rheumatic fever, but most of the patients were diagnosed few months before their referral and had taken only one or two doses.

## Conclusion

Our study showed that in our population, rheumatic mitral stenosis is common in the first and early second decades of life, with a rapid clinical progression to symptoms and disability. Echocardiography-based screening programs are needed to determine the true prevalence of rheumatic valvular disease amongst our childhood population, and in course, strengthen the commitment to primary and secondary prevention programs.

Our study has a number of limitations. This is a hospital-based study. It is likely that only advanced symptomatic cases are referred to us, representing the proverbial tip of a very great iceberg. Our patients are not likely to be representative of the full extent of the disease burden at the community level. Additionally, our study is a retrospective in design, and many important variables were incomplete, making comprehensive analysis of risk factors difficult. Nevertheless, this study provides new insights into the severity, complexity, and rapid progression of rheumatic valvular heart disease amongst young Ethiopians, and begs for a more comprehensive population analysis.

## Abbreviations

RHD: Rheumatic heart disease; NYHA: New York Heart Association; WHO: World Health Organization; MVA: Mitral valve area; MS: Mitral stenosis; GLA: Giant left atrium.

## Competing interests

The authors have no conflict of interest to declare.

## Authors’ contributions

HT reviewed literature, prepared the proposal, collected data and wrote the draft manuscript. WM participated in the design of the study and assisted in statistical analysis of the data. ET assisted in formulating the study question, reviewed literature and wrote the final version of the manuscript in its current form. All authors have read and approved the final version of the manuscript.

## Authors’ information

HT is a pediatrician working in the department of Pediatrics & Child Health of the School of Medicine of Hawassa University. He was a resident in the department of Pediatrics & Child Health of the School of Medicine, Addis Ababa University from November 2009-December 2012. WM is a Statistician at the School of Public Health, Addis Ababa University. ET is a Consultant Pediatric Cardiologist at the School of Medicine of Addis Ababa University and the Cardiac Center in Addis Ababa.

## Pre-publication history

The pre-publication history for this paper can be accessed here:

http://www.biomedcentral.com/1471-2261/13/95/prepub
